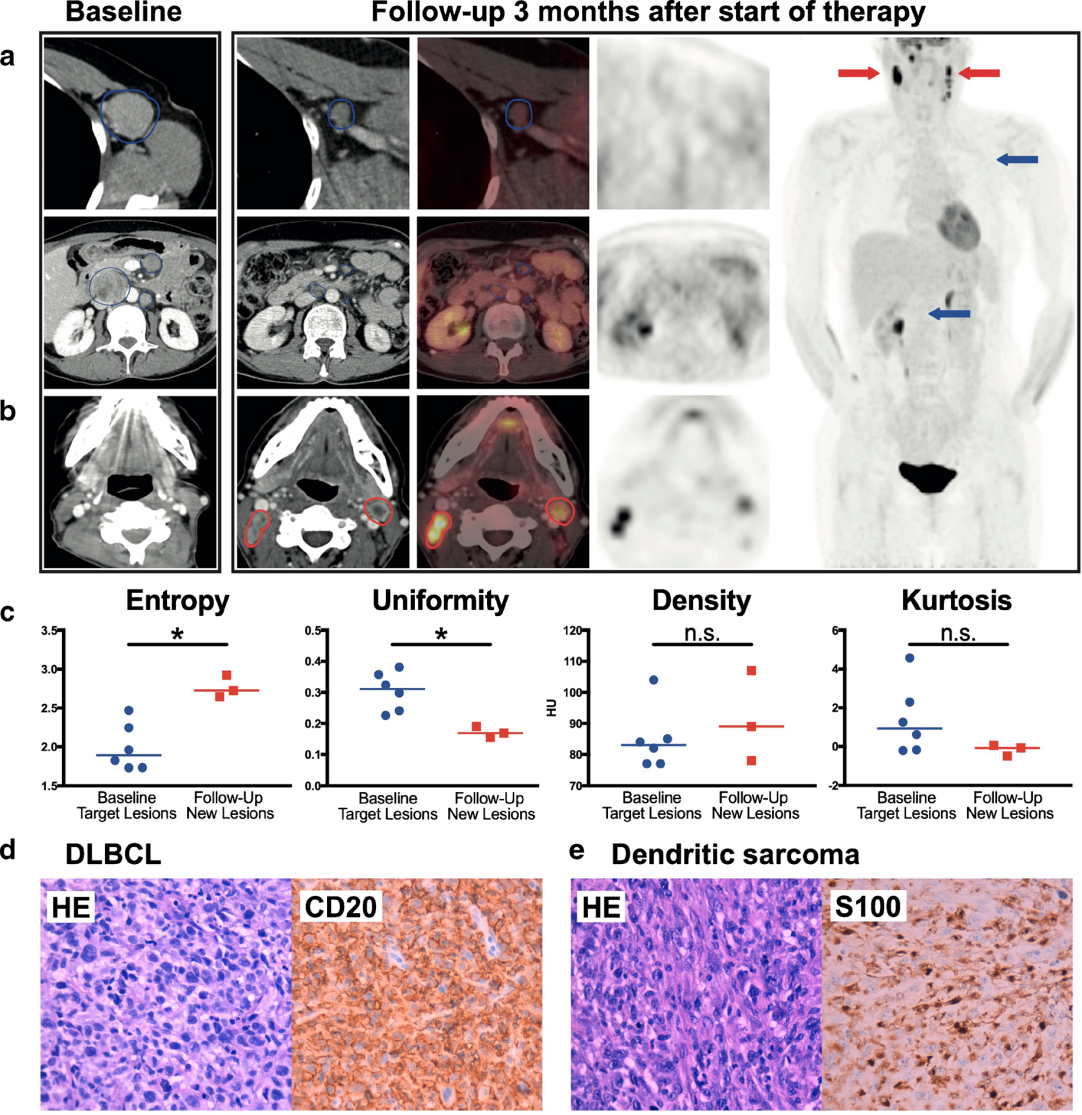# Transformation of diffuse large B cell lymphoma into dendritic sarcoma under CAR T cell therapy detected on ^18^F-FDG PET/CT

**DOI:** 10.1007/s00259-020-05000-9

**Published:** 2020-08-19

**Authors:** Michael Winkelmann, Kai Rejeski, Marcus Unterrainer, Christian Schmidt, Michael Ruzicka, Jens Ricke, Martina Rudelius, Marion Subklewe, Wolfgang G. Kunz

**Affiliations:** 1 Department of Radiology, University Hospital, LMU Munich, Marchioninistr. 15, 81377 Munich, Germany; 2 Department of Medicine III, University Hospital, LMU Munich, Munich, Germany; 3 Department of Pathology, University Hospital, LMU Munich, Munich, Germany

Chimeric antigen receptor (CAR) T cell immunotherapy uses patient-derived tumor antigen-directed T cells for targeted elimination of cancer cells [1]. The most common form applies modified T cells expressing a CAR specific for the CD19 antigen to treat relapsed or refractory lymphoma [2] and leukemia [3].

We present a 60-year-old female patient with refractory diffuse large B cell lymphoma (DLBCL) who underwent CAR T cell therapy. During treatment, all lesions decreased in size with a complete metabolic response (Deauville score 1) in ^18^F-FDG PET/CT imaging obtained 3 months after CAR T cell infusion (A). At the same time, multiple newly enlarged and hypermetabolic cervical lymph nodes (SUVmax value = 31) were detected in a previously unaffected location (B). These new lesions (red circles and arrows) showed a morphological dedifferentiation with a large central hypodensity compared with nodal DLBCL target lesions at baseline CT (blue circles and arrows). This was also reflected by differences in the radiomic features entropy and uniformity (C). These circumstances triggered a repeat histological workup that determined the transformation of the DLBCL (D; high CD20 expression) into a sarcoma of the dendritic cells (E; high S100 expression) without residual lymphomatous tissue. Based on high PD-L1 expression, checkpoint inhibition with pembrolizumab was initiated.

Rare cases of transformation into histiocytic and dendritic cell neoplasms have been reported in patients with follicular lymphoma and DLBCL [4, 5]. This case underlines the diagnostic potential in the interlesional comparison of morphologic and metabolic features to raise the suspicion of clonal dedifferentiation. Future studies that correlate radiomic features from imaging and pathologic features from biopsies may not only lead to diagnostic improvements but also a better understanding of tumor biology in patients undergoing CAR T cell therapy.